# Electrochemical Detection of Carbon Steel Corrosion Induced by Fermentative Bacteria From Natural Gas Transmission Lines

**DOI:** 10.1111/1758-2229.70058

**Published:** 2024-12-10

**Authors:** Joshua A. Davis, Sai Prasanna Chinthala, Chelsea N. Monty‐Bromer, John M. Senko

**Affiliations:** ^1^ Department of Biology The University of Akron Akron Ohio USA; ^2^ Integrated Biosciences The University of Akron Akron Ohio USA; ^3^ Department of Chemical and Biological Engineering Cleveland State University Cleveland Ohio USA; ^4^ Department of Geosciences The University of Akron Akron Ohio USA

**Keywords:** acid production, fermenter, microbially influenced corrosion, natural gas

## Abstract

The metabolic potential and corrosive activities of a fermentative bacterial enrichment culture from a natural gas transmission line were characterised. Three metagenome‐assembled genomes (MAGs) attributable to *Cytobacillus*, *Lacrimispora* and *Staphylococcus* spp. were obtained. These MAGs hosted genes involved in the fermentation of carbohydrates to organic acids, which was reflected in the acidification of the growth medium by the culture. To evaluate the corrosive activities of the culture, it was incubated in a split chamber‐zero resistance ammetry (SC‐ZRA) format. This involved deploying carbon steel coupons immersed in liquid medium in opposing chambers of an electrochemical cell. Measurement of current between the coupons indicated the extent and mechanism of corrosion. When the enrichment culture was added to one side of an SC‐ZRA incubation with bicarbonate‐buffered medium, pH change and corrosion were minimal. In bicarbonate‐free medium, the culture acidified the medium, induced electron transfer from the uninoculated chamber to the inoculated chamber, and caused mass loss. These results indicate that fermenter‐induced microbially influenced corrosion (MIC) is due to localised fluid acidification, inducing anodic reactions on the metal surface exposed to the microorganisms and mass loss of the non‐exposed metal.

## Graphical abstract

The corrosive activities of a fermentative enrichment culture from natural gas line pigging sludge were analysed using an electrochemical technique known as zero‐resistance ammetry. Corrosive activity was detected through current measurements, indicating that corrosion occurred due to the production of acidic fermentation products. The findings suggest that acid‐induced anodic conditions near bacterial activities can cause corrosion.

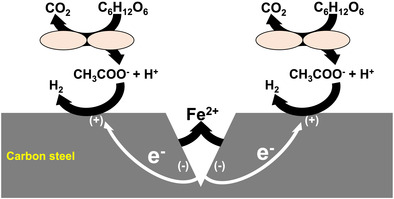

## 1 Introduction

Microbially influenced corrosion (MIC) of fluid and gas handling equipment and pipelines is a pressing problem in a variety of industrial settings, including food and beverage, wastewater treatment, and oil and gas (Babu et al. 2006; Di Franco et al. 2021; Khan, Hussain, and Djavanroodi 2021; Kokilaramani et al. 2021). In the oil and gas industry, MIC is often appropriately attributed to the activities of sulphate‐reducing bacteria (SRB) (e.g. Enning and Garrelfs 2014; Little et al. 2020; Vigneron et al. 2016; Vigneron, Head, and Tsesmetzis 2018). However, oil and gas processing and transport facilities host metabolically diverse microbial communities (e.g. Duncan et al. 2009; Gieg, Fowler, and Berdugo‐Clavijo 2014; Vigneron et al. 2016; Vigneron, Head, and Tsesmetzis 2018). In most settings, the microbial communities that carry out corrosion are complex assemblages of organisms, and in many cases, these involve cooperative interactions between fermentative and SRB (Davidova et al. 2012; Vigneron et al. 2016; Vigneron, Head, and Tsesmetzis 2018). Fermentative bacteria that are encountered in oil and gas handling systems may enhance corrosion by degrading relatively complex organic substrates to H_2_ and/or organic acids, thus facilitating the activities of corrosive SRB (Babu et al. 2006; Duncan et al. 2009; Gieg, Fowler, and Berdugo‐Clavijo 2014; Lyles et al. 2014; Neria‐González et al. 2006; Vigneron et al. 2016; Vigneron, Head, and Tsesmetzis 2018).

Alternatively, fermentative organisms (sometimes referred to as acid‐producing bacteria) in these communities may contribute to the overall process of MIC via production of acidic byproducts (Di Franco et al. 2021; Duncan et al. 2009; Gieg, Fowler, and Berdugo‐Clavijo 2014; Gu 2014; Vigneron et al. 2016; Vigneron, Head, and Tsesmetzis 2018; Xu, Li, and Gu 2016). It has been proposed (Gu 2014; Xu, Li, and Gu 2016) that a major mechanism of MIC caused by fermentative bacteria occurs under anoxic conditions when organic acid fermentation products (e.g. formic, acetic, lactic acids) accumulate under low‐sulphate conditions. Here, organic acids (depicted as acetic acid for simplicity; Suflita, Phelps, and Little 2008) are reduced by Fe^0^ ((R1), (R2), (R3)).

(R1) $${\mathrm{Fe}}^{0}+{\text{2CH}}_{3}\text{COOH}\to {\mathrm{Fe}}^{2+}+H_{2}+{\text{2CH}}_{3}{\mathrm{COO}}^{-}$$

(R2) $${\text{2CH}}_{3}\text{COOH}\to {\text{2CH}}_{3}{\mathrm{COO}}^{-}+{\text{2H}}^{+}$$

(R3) $${\text{2H}}^{+}+{\mathrm{Fe}}^{0}\to {\mathrm{Fe}}^{2+}+H_{2}$$

Whilst it is not clear if the protonated acid serves as the electron donor for Fe^0^ oxidation (R1) or if dissociated protons (R2) are the oxidants of Fe^0^ (R3), the ultimate outcome is oxidation of Fe^0^ with H_2_ production.

To test the hypothesis that fermentative acid production enhances corrosion, we enriched for fermentative microorganisms from a natural gas transmission line and examined the composition and metabolic potential of the enriched organisms. We then used a split chamber‐zero resistance ammetry (SC‐ZRA) technique to characterise the corrosive activities of the enrichment culture under chemical conditions approximating those of a gas transmission line. In SC‐ZRA, two shorted carbon steel electrodes are deployed in separate, liquid‐containing chambers that are connected by a semipermeable membrane or salt bridge (Miller et al. 2018, 2016, 2020). One chamber can be inoculated, and current is measured between the electrodes. In this configuration, SC‐ZRA mimics heterogeneous metal coverage by microorganisms, which can lead to corrosion (Miller et al. 2023, 2020). The magnitude of the current can be indicative of the extent of corrosion, whilst the direction of current can indicate where anodic and cathodic processes leading to corrosion are occurring (i.e. in the inoculated or uninoculated side of the chamber; Miller et al. 2023, 2020).

## 2 Experimental Procedure

### 2.1 Bacterial Cultivation

Natural gas transmission line pigging sludge was used as an inoculum for a fermentative enrichment culture using a medium designed to mimic the high dissolved solids content of aqueous solutions associated with oil and gas extraction, processing and transport (Chinthala et al. 2024). The brine‐based enrichment medium contained 20 mM glucose as a carbon and energy source, and 350 mM NaCl, 30 mM NaHCO_3_, 25 mM CaCl_2_, 15 mM MgCl_2_, 2 mM KCl, 0.05 mM K_2_HPO_4_, 0.07 g/L yeast extract, vitamins and trace metals (Tanner 2007). The initial enrichment culture was established using a 10% inoculum of pigging sludge in the brine‐based medium. The enrichment culture was maintained through ~15 transfers before it was used in corrosion experiments. In preparation for SC‐ZRA experiments, the enrichment culture was grown in 1 L of the brine‐based medium to stationary phase. Cells were harvested by centrifugation, then washed and resuspended three times with the brine‐based medium. The concentrated cell suspension was then added to incubations to achieve an *A*_600_ of 1.

### 2.2 SC‐ZRA Incubations

Carbon steel (UNS G10180) working electrodes (exposed surface area 652 mm^2^) were prepared for SC‐ZRA experiments by polishing them with progressively finer SiC papers of 240, 320, 400 and 600 grits as described in ASTM standard E1558‐09 (ASTM 2014). After obtaining an initial mass, the carbon steel coupons were sterilised by placing them in the SC‐ZRA apparatus, replacing air with N_2_, and placing the SC‐ZRA apparatus (with the steel coupons) in an oven at 160°C for 4 h. This process sterilises the metal, whilst minimising any alterations to the metal surface, which occurs during other common sterilisation approaches (e.g. autoclaving) (Giai et al. 2016). Brine‐based medium (450 mL; described above) was added to both the chambers of the split cell in an anaerobic chamber (Coy Laboratory Products, Grass Lake, MI) and sealed. Where appropriate, sodium bicarbonate was omitted from the brine‐based medium. The two chambers of the SC‐ZRA were connected by a salt bridge filled with saturated potassium chloride solution. The carbon steel working electrodes (referred to as WE1 and WE2) were included in the respective chambers with a saturated calomel electrode (SCE) as reference electrode in the chamber with WE1. After completing the SC‐ZRA setup, the headspace of the chambers was replaced with filter‐sterilised 80:20 N_2_:CO_2_ or N_2_ for incubations with and without sodium bicarbonate respectively. Where appropriate, the WE1 chamber was inoculated with the enrichment culture (called FE1), which was prepared as described above. Current and potential were measured using a BioLogic potentiostat/galvanostat (VSP‐300; BioLogic Science Instruments, Seyssinet‐Pariset, France) every 15 min. In this configuration, a positive current represents electron transfer from WE1 to WE2 and a negative current represents electron transfer from WE2 to WE1. Samples were periodically obtained from both chambers to measure pH, glucose and organic acid concentrations (described below). At the conclusion of the incubations, the WEs were removed subjected to mass loss analysis (described below).

### 2.3 Nucleic Acid Sequencing and Analysis

In preparation for nucleotide sequencing, genomic DNA was extracted from samples using the DNeasy PowerLyzer PowerBiofilm Kit (Qiagen, Germantown, MD). For 16S rRNA gene sequencing, PCR amplification of the 16S rRNA V4 region using the primers 515F (5′‐GTG CCA GCM GCC GCG GTA A‐3′) and 806R (5′‐GGA CTA CHV GGG TWT CTA AT‐3′) (Caporaso et al. 2011) was performed using unique barcodes along with Illumina adapter sequences (Integrated DNA Technologies, Coralville, IA). PCR was performed using a Mastercycler Nexus Gradient (Eppendorf, Enfield, CT) with a 3 min 94°C hot start, followed by 30 cycles of: denaturation at 94°C for 45 s, annealing at 50°C for 60 s and then a 72°C extension for 90 s, followed by a final extension step at 72°C for 10 min. The PCR products were gel purified and quantified using a Qubit dsDNA HS Assay Kit (Life Technologies, Waltham, MA). Samples were sequenced on an Illumina MiSeq with paired end 250 bp reads. Taxonomic assignments were made by processing raw sequences through QIIME 22020.2 (Bolyen et al. 2019) using the q2 quality filter and deblur plugins to quality filter (Amir et al. 2017; Bokulich et al. 2013) and the q2‐feature‐classifier (Bokulich et al. 2018; Pedregosa et al. 2011) classify sklearn taxonomy classifier against the Greengenes 18_8 99% OTUs reference sequences to assign taxonomies to the raw sequences (Bokulich et al. 2018; McDonald et al. 2012).

For metagenomic DNA sequencing, libraries were prepared using Illumina DNA Prep (M) Tagmentation library preparation kit (Illumina, San Diego, CA) (Haendiges et al. 2020) following the manufacturer's instructions. Initial DNA concentrations were evaluated using the Qubit dsDNA HS Assay Kit (Life Technologies, Waltham, MA). The DNA was then cleaned using DNEasy PowerClean Pro Cleanup Kit (Qiagen, Germantown, MD) and the DNA concentration was analysed again. Libraries were prepared for each sample by using 30–50 ng DNA which underwent simultaneous fragmentation and addition of adapter sequences. These adapters were utilised during a limited‐cycle PCR, in which unique indices were added to the sample. Following library preparation, libraries were pooled in equimolar ratios of 0.6 nM and sequenced paired end for 300 cycles using the NovaSeq 6000 system (Illumina, San Diego, CA).

Raw metagenomic reads were assessed, trimmed, assembled, quality assessed, binned into putative genomes, quality assessed again, and quality‐filtered bins were extracted as assemblies, and taxonomies of both assemblies and raw reads were assigned using Kbase 2.6.4 (Arkin et al. 2018). The following applications were used for each step, respectively: FastQC (v0.11.9), Trimmomatic (v0.36) (Bolger, Lohse, and Usadel 2014), metaSPAdes (v3.15.3) (Nurk et al. 2017; Prjibelski et al. 2020), CheckM (v1.0.18) (Parks et al. 2015), MetaBAT2 Contig Binning (v1.7) (Kang et al. 2019), CheckM (v1.0.18) (Parks et al. 2015), extract bins as assemblies from BinnedContigs (v1.0.2) (Arkin et al. 2018) and GTDB‐Tk (v1.7.0) (Chaumeil et al. 2019; Parks et al. 2018, 2020). Kaiju was used to assign taxonomies using protein‐level classifications to sequencing reads by comparing read sequences to the NCBI RefSeq database of completely assembled bacterial, archaeal and viral genomes (Menzel, Ng, and Krogh 2016; Ondov, Bergman, and Phillippy 2011). Metagenome and 16S rRNA sequence data is available at NCBI under BioProject PRJNA1058620.

### 2.4 Analytical Techniques

Corrosion rates of WEs were determined by weight loss analysis (WLA) using ASTM method G1‐03 (ASTM 2011). Samples were rinsed with deionised water, wire brushed, and then, immersed in Clarke's reagent (0.26 M SnCl_2_ and 0.07 M Sb_2_O_3_ in 12.1 M HCl) for 30 s to remove surface oxides. After the Clarke's reagent treatment, the coupons were rinsed with DI water, dried and weighed. These steps were repeated until no mass was lost between wash cycles, indicating that all oxides were removed (ASTM 2011). The total mass loss was recorded indicating the physical weight that was lost due to corrosivity of the environment. Corrosion rate was calculated using the following equation:

(1) $$\mathrm{CR}=\frac{W\times K}{D\times A\times t}$$

where CR represents the corrosion rate in mm/yr, *K* (8.76 × 10^4^) is a dimensionless constant, *W* is the mass loss in grams, *A* is the exposed surface area of the coupon in cm^2^, *T* is exposure time in hours and *D* is the density of carbon steel UNS G10180 in g/cm^3^ (ASTM 2011). Subsequently, the corrosion rate ratio (CRR) was calculated by dividing the rate of corrosion of WE1 by the rate of corrosion of WE2. Biotic corrosion rates were calculated based on the difference in corrosion rates of WE1 and WE2 that was a result of microbiological activity (i.e. not abiotic corrosion observed in sterile controls). Based on Faraday's law, mass loss caused by microorganisms was predicted using the following equation:

(2) $$∆m=\frac{\int \text{Qdt}\;a}{\mathrm{nF}}$$

where Δ*m* is the mass loss, *I* is the current produced from the SC‐ZRA experiment where *Q* is charge passed, *t* is the time, *a* is the atomic mass, *n* is the valence change and *F* is the Faraday's constant. The predicted corrosion rate was then calculated using Equation (1). Acetate was quantified by high‐performance liquid chromatography by using an Agilent 1200 HPLC system (Agilent Technologies Inc., Santa Clara, CA) equipped with an Aminex HPX‐87H column (300 mm × 7.8 mm; Bio‐Rad Laboratories Inc., Hercules, CA) with UV (254 nm) detection (SPD‐10A). A mobile phase of 0.008 N H_2_SO_4_ was used at a flow rate of 0.6 mL/min. pH was measured using Orion 370 PerpHect pH meter (ThermoFisher Scientific, Waltham, MA).

## 3 Results and Discussion

### 3.1 Bacterial Enrichment and Culture Composition

**FIGURE 1:**
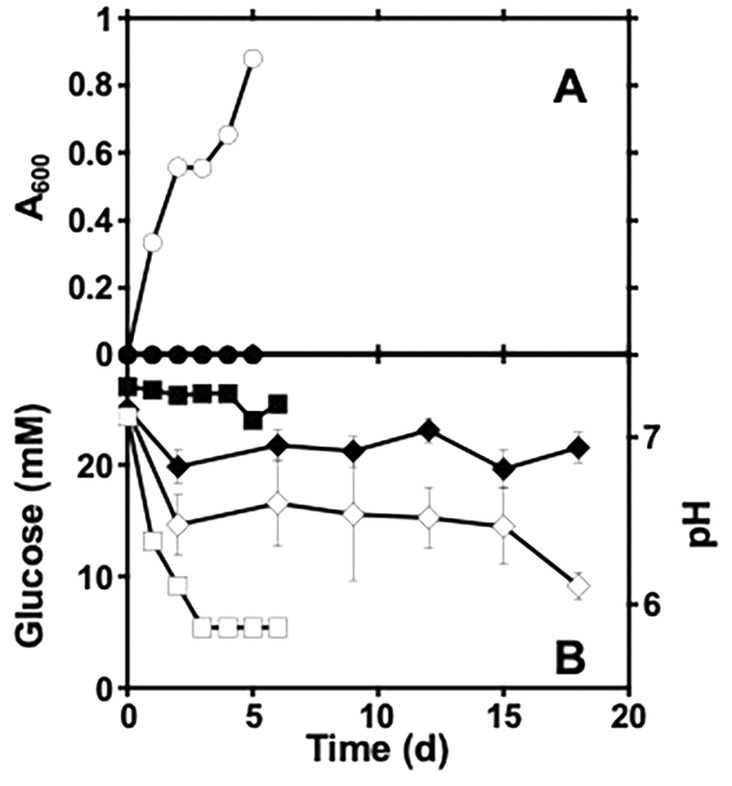
Growth (as indicated by *A*_600_; circles in panel A), glucose concentrations and pH (diamonds and squares, respectively in panel B) of FE1 after 10 transfers. Filled and open shapes represent values in uninoculated and inoculated media, respectively. Error bars represent standard deviations of duplicate measurements.

In previous work, we reported the enrichment and corrosive activities of sulphate‐reducing microorganisms from natural gas line pigging sludge (Chinthala et al. 2024). To enrich for fermentative microorganisms from the same sample, we used it to inoculate medium that was designed to mimic the aqueous chemistry of the pipeline interior (e.g. 350 mM NaCl). Potential terminal electron acceptors (i.e. oxygen and sulphate) were omitted from the medium and glucose was provided as a fermentative substrate. The enrichment culture (referred to as FE1) was maintained for over 25 transfers. Growth (as indicated by optical density) occurred concurrently with glucose depletion and the culture reached a maximum *A*_600_ of 1 after 5 days (Figure 1A,B). Growth and glucose metabolism caused a decrease in pH of the medium (Figure 1B), suggesting that organic acids were products of glucose fermentation. A 16S rRNA partial gene‐based survey of the enrichment culture after 15 transfers revealed that it was predominantly composed of Bacillota (54%) and Synergistota (42%).

### 3.2 Metagenomic Analysis of Enrichment Culture

To determine the metabolic potentials of the organisms in the FE1 enrichment culture, we analysed the metagenome that we recovered from it after 25 transfers. Three metagenome‐assembled genomes (MAGs) were obtained with coverage of 98%, 100% and 99%, with the largest contig length being 13,261,126 nucleotides. To assess the genomes for completeness and contamination, we used the CheckM application. CheckM can generate clade‐specific marker genes sets for each bin and reports the taxonomic resolution possible for each bin, which is known as a marker lineage (Parks et al. 2015). CheckM indicated that two of our MAGs had contamination percentages of 1.58% (MAG 1), 1.99% (MAG 2) and 0% (MAG 3). It should be noted that indication of contamination in CheckM are underestimates, and could be responsible for less specificity in the marker lineage identification (Parks et al. 2015). Because our genomes have high completeness, the estimated extents of contamination of the MAGs are likely accurate. To taxonomically assign our MAGs, we used the Genome Taxonomy Database Toolkit (GTDB‐Tk), which uses the bacterial and archaeal reference trees, multiple sequence alignments and taxonomy provided through the GTDB (Chaumeil et al. 2019). MAGs were taxonomically assigned to *Cytobacillus oceanisediminis* (MAG 1), *Lacrimispora amygdalina* (MAG 2) and *Staphylococcus epidermidis* (MAG 3), all three of which are affiliated with the Bacillota, which was the predominant phylum in the partial 16S rRNA gene‐based survey. These three taxa appear to be the predominant components of the enrichment culture, because most raw sequence reads were assigned to *C. oceanisediminis* (13%), *L. amygdalina* (33%) and *S. epidermidis* (9%), using Kaiju (Menzel, Ng, and Krogh 2016; Ondov, Bergman, and Phillippy 2011), with 30% unassignable at the species level, and the remaining sequences assigned to taxa comprising less than 2% of the sequence reads. Indeed, when we decreased the completeness threshold to 60% and increased the contamination threshold to 40%, only the MAGs attributable to *C. oceanisediminis* (13%), *L. amygdalina* (33%) and *S. epidermidis* (9%) could be obtained.

*C. oceanisediminis* is an aerobic, spore‐forming, mesophilic bacterium found in marine sediment (Lee et al. 2012). *Lacrimispora* spp. are cosmopolitan Gram‐positive anaerobes that has been isolated from wastewater treatment plants sludge and animal hosts (Cornick et al. 1994; Palop et al. 1989; Parshina et al. 2003), whilst *S. epidermidis* is a Gram‐positive commensal bacterium that has been isolated from soil, water and various surfaces in non‐human settings (Gerken, Wiegner, and Economy 2022; Kloos 1980; Prussin II and Marr 2015). The recovery of these taxa from the gas pipeline is reflective of a physicochemical setting that is impacted by human activities with high dissolved solids (Brown, Brown, and Senko 2012; Martin et al. 2021; Thapaliya et al. 2017). We screened MAGs for enzymes involved in carbohydrate metabolism, particularly those yielding organic acid byproducts that could cause carbon steel corrosion (Madirisha, Hack, and van der Meer 2022). All three MAGs contained complete Emden‐Meyerhof‐Parnas pathways, and potential pathways of fermentation product formation by each of the three MAGs are shown in Table 1.

**TABLE 1:** Enzymes involved in fermentative reactions detected in MAGs attributed to *C. oceanisediminis*, *S. epidermidis* and *L. amygdalina*.

Metabolism and enzymes	MAG 1 (*C. oceanisediminis*)	MAG 2 (*L. amygdalina*)	MAG 3 ( *S. epidermidis* )
Acetate production			
Phosphate acetyl transferase	+	+	+
Acetate kinase	+	+	+
Ethanol production			
Acetaldehyde dehydrogenase	+	+	−
Alcohol dehydrogenase	−	+	+
Lactate production			
Lactate dehydrogenase	+	+	+
D‐lactate dehydrogenase	−	−	+
Succinate production			
Malate dehydrogenase (quinone)	+	−	+
Malate dehydrogenase (oxaloacetate‐decarboxylating)	+	+	+
Malate dehydrogenase (oxaloacetate‐decarboxylating) (NADP^+^)	+	−	+
Fumarate hydratase	+	−	+
Formate production			
Formate acetyltransferase (pyruvate‐formate lyase)	−	+	+
Pyruvate‐formate lyase activating enzyme	−	+	+
Butyrate production			
Pyruvate‐ferredoxin oxidoreductase	−	+	−
Acetyl‐CoA C‐acetyltransferase	+	+	+
3‐Hydroxybutyryl‐CoA dehydrogenase	+	−	−
4‐Hydroxybutyryl‐CoA dehydratase	−	+	−
Butyryl‐CoA dehydrogenase	+	−	−
Phosphotransbutyrylase	+	−	−
Butyrate kinase	+	+	−

Metabolic characterisation of a strain of *C. oceanisediminis* (H2^T^) indicated it metabolised a variety of carbohydrates under oxic conditions, but anaerobic fermentation products were not evaluated (Zhang et al. 2010). Genomic characterisation of *C. oceanisediminis* 2691 revealed phosphotransferase systems for glucose metabolism and pathways for mixed acid fermentation of glucose to acetate, ethanol and lactate (Lee et al. 2012). The MAG attributable to *C. oceanisediminis* in our enrichment can produce acetate, lactate and succinate from glucose fermentation, but pathways for ethanol, formate and butyrate production are absent or incomplete (Table 1). *Lacrimispora* (formerly *Clostridium*) spp. have been isolated from sewage sludge and industrial mudpits, and can ferment carbohydrates to ethanol, acetate, lactate and butanol (Jin et al. 2023; Parshina et al. 2003). The *L. amygdalina*‐attributable MAG in our enrichment contained incomplete pathways for fermentation of glucose to succinate and butyrate, but included genes necessary for glucose fermentation to acetate, ethanol, lactate and formate (Table 1). As human commensals, Staphylococci are encountered in human‐impacted and built environments (de Sousa et al. 2017; Gerken et al. 2022; Kloos 1980; Prussin II and Marr 2015). *S. epidermidis* strains ferment glucose to lactate, but may produce, acetate, formate and ethanol (Pedroza‐Dávila et al. 2020; Sivakanesan and Dawes 1980). The *S. epidermidis* ‐attributable MAG harboured complete pathways for fermentation of glucose to acetate, ethanol, lactate, succinate and formate, but the pathway for butyrate production was incomplete (Table 1). These observations indicate acidification of medium during growth of FE1 was attributable to the accumulation of organic acids by the three MAGs recovered from the enrichment, and these activities could influence corrosion in steel pipelines.

### 3.3 Corrosive Activities of FE1 Under Fermentative Conditions

**FIGURE 2:**
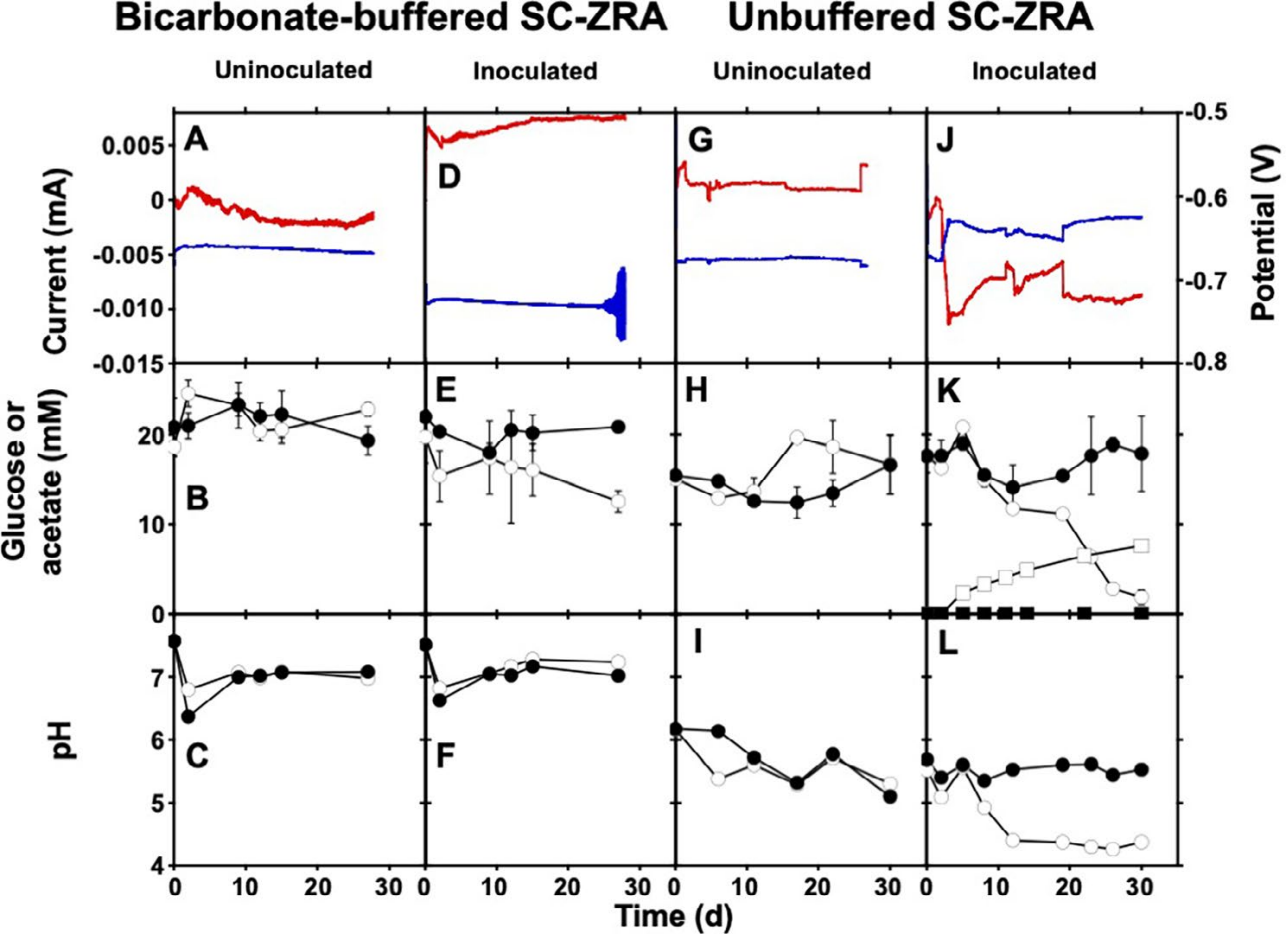
Current and potential (A, D, G and J), glucose and acetate concentrations (B, E, H and K) and pH (C, F, I and L) in SC‐ZRA incubations with bicarbonate buffering that were uninoculated (A–C) or inoculated with FE1 (D–F) and without bicarbonate buffering that were uninoculated (G–I) or inoculated with FE1 (J–L). Current and potential are in red and blue, respectively in panels A, D, G and J. pH and glucose concentrations are depicted by open (WE1 chamber) and closed (WE2 chamber) circles in their respective panels. Acetate concentrations are depicted in panel K using open (WE1 chamber) and closed (WE2 chamber) squares.

To evaluate the corrosive activities of our culture, we carried out a series of SC‐ZRA incubations. In uninoculated SC‐ZRA incubations with bicarbonate‐containing synthetic brine, current was initially slightly positive, but no glucose metabolism or pH change were detected (Figure 2A–C). Consistent with this lack of activity was the similarity in rates of corrosion of WE1 and WE2 (as indicated by CRR) and low biotic corrosion rate (difference in corrosion rate between WE1 and WE2; Table 2). When synthetic brine was inoculated with FE1, glucose was metabolised but no decrease in pH was observed in either the WE1 or WE2 chamber (Figure 2B,C). Positive current was observed, indicating minimal transfer of electrons from WE1 to WE2. Greater corrosion was observed on WE1 compared to WE2 (as indicated by corrosion rate and CRR; Table 2). Additionally, the biotic corrosion rate increased (Table 2). The positive current and increased biotic corrosion rate indicates that direct exposure of the WE1 electrode to FE1 minimally enhanced its corrosion rate in the bicarbonate‐buffered system.

**TABLE 2:** Treatment corrosion rates or WE1 and WE2, corrosion rate ratios (CRR) biotic corrosion rates (amount of corrosion induced by microbial activities and not abiotic) and predicted corrosion rates (based on current; Equation 2).

Treatment	Overall corrosion rate (mpy)	Overall corrosion rate ratio (WE1:WE2)	Actual biotic corrosion rate (mpy)	Predicted biotic corrosion rate (mpy)
With bicarbonate, uninoculated	WE1 3.9 WE2 3.9	1.0	0.0	0.2
With bicarbonate, WE1 chamber inoculated	WE1 4.7 WE2 3.6	1.3	1.1	1.0
Without bicarbonate, uninoculated	WE1 4.5 WE2 4.9	0.9	0.4	0.2
Without bicarbonate, WE1 chamber inoculated	WE1 1.4 WE2 2.7	0.5	1.3	1.1

We hypothesised that the bicarbonate in the synthetic brine was limiting pH change, and therefore inducing microbial corrosion of the carbon steel coupons. To test this hypothesis, we conducted a second series of SC‐ZRA incubations without bicarbonate in the brine. In SC‐ZRA incubations without bicarbonate buffering, and no FE1 inoculation, the starting pH was lower than incubations with bicarbonate added, and it changed minimally over the course of the incubations (Figure 2I). Glucose was not consumed, and no organic acid accumulation was observed (Figure 2H). Little current was observed between the WEs, indicating that they were in electrochemical equilibrium, which is consistent with the CRR of 0.9 and a low biotic corrosion rate of 0.4 (Table 2). When FE1 was added to the WE1 chamber of an SC‐ZRA incubation, glucose metabolism proceeded in the WE1 chamber, accompanied by accumulation of acetic acid (Figure 2K). The accumulation of organic acid resulted in a pH decrease to approximately 4.5 in the WE1 chamber, but not the WE2 chamber (Figure 2L). We were unable to detect succinate, butyrate, lactate or formate as products of glucose metabolism during the SC‐ZRA incubations. All three MAGs recovered from the enrichment were capable of fermentation of glucose to acetate, which is consistent with the observation of acetate accumulation. However, low pH (i.e. < 5) may modify the fermentation product profiles of *Lacrimispora* spp. (Jin et al. 2023). The pH decrease enhanced the development of negative current, indicating electron transfer from WE2 to WE1 (Figure 2J). This pattern of electron transfer is consistent with cathodic reactions (R1)–(R3) occurring in the WE1 chamber, and the anodic reaction (R4) occurring in the WE2 chamber.

(R4) $${\mathrm{Fe}}^{0}\to {\mathrm{Fe}}^{2+}+{\text{2e}}^{-}$$

Here, the decrease in pH provides a driving force for hydrogen generation and the Fe^0^ corrodes on the WE2 to maintain equilibrium. The CRR of the WEs and biotic corrosion rate was consistent with this negative current (Table 2), where WE2 (the anode) was corroding more rapidly than WE1 (the cathode).

Although the corrosion rates in SC‐ZRA incubations with FE1 and with and without bicarbonate buffer are generally lower than those of the uninoculated controls, there is minimal to no current flow and no evidence of microbial corrosion. The SC‐ZRA method focuses on assessing the overall corrosion driven by microbial activity and is primarily concerned with the increased biotic corrosion rate resulting from the inoculation with FE1. The SC‐ZRA approach allows us to determine corrosion caused by microbiological activities despite potential metallurgical differences between coupons, which could confound direct mass loss‐based corrosion rate calculations. Comparing the actual and predicted biotic corrosion rates (Table 2) indicates the success of the SC‐ZRA technique to accurately predict the biotic corrosion rate with an accuracy of approximately 90% for samples inoculated with FE1.

### 3.4 Implications for MIC by Fermentative Microorganisms

Fermentative bacteria are encountered in a variety of oil/gas handling and other industrial settings and are important mediators of organic carbon degradation (Di Franco et al. 2021; Duncan et al. 2009; Gieg, Fowler, and Berdugo‐Clavijo 2014; Gu 2014; Vigneron et al. 2016; Vigneron, Head, and Tsesmetzis 2018). This can include organisms that ferment carbohydrates and other types of non‐hydrocarbon organic compounds (Duncan et al. 2009; Gieg, Fowler, and Berdugo‐Clavijo 2014; Vigneron et al. 2016; Vigneron, Head, and Tsesmetzis 2018). Whilst hydrocarbons are generally the predominant organic substrate in oil and gas, a far wider variety of organic compounds may be metabolic substrates, including compounds used in oil and gas extraction, transport and processing, as well as microbial cross‐feeding (Annuk and Moran 2010; Fritts, McCully, and McKinlay 2021; Li et al. 2023; Luek and Gonsior 2017). In any case, organic acids are important products of organic carbon metabolism and may cause corrosion.

The results of this work illustrate the dynamics of MIC under fermentative conditions. First, we have shown that localised acidification induces cathodic reactions (R1 and R2), thus inducing mass loss via R4. The SC‐ZRA approach allowed us to mimic the conditions of localised fermentative activity on a metal surface, which induces localised corrosion. Second, we show that the activities of fermentative bacteria can cause corrosion independently of their metabolic relationships with SRB. Third, the work illustrates the influence of pH and buffering on fermenter‐induced MIC. In oil and gas extraction and processing settings, pH and alkalinity can vary dramatically. In most cases, the pH and alkalinity of brines is influenced by the dynamics of the carbonic acid system, although carboxylic acids can also influence the alkalinity of brines (Maskari et al. 2020; Sanchez‐Rosario and Hildenbrand 2022; Thyne and Brady 2016; Zhang et al. 2019). pH of produced waters typically falls in a range of 4–8, but extremes below 2 and above 8 have been reported (Alley et al. 2011; Emam, Moawad, and Aboul‐Gheit 2014; Li 2013). Alkalinities can range from 0 to 5000 meq/L (Sanchez‐Rosario and Hildenbrand 2022; Zhang et al. 2019) and are a reflection of the oil/gas host rock and associated brine. Brines/production waters from carbonate‐rich reservoirs will have higher alkalinities (Maskari et al. 2020; Thyne and Brady 2016). Post‐extraction degassing of CO_2_ will also influence pH and alkalinity (de Paula Cosmo et al. 2022). The work we present here illustrates how variability in pH and buffering of brines and production waters could control the extents of corrosion by fermentative microorganisms from gas transmission lines.

## 4 Conclusion

This work illustrates the role of fermentative bacterial activities in MIC that is independent of SRB. When production of organic acid metabolites decreases the pH of solutions, corrosion occurs via the development of localised anodes and cathodes on metals. In the case of fementer‐induced MIC, acid production causes the development of anodic conditions in proximity to the bacterial activities.

## Author Contributions

**Joshua A. Davis:** investigation, writing – original draft, formal analysis, methodology, visualization. **Sai Prasanna Chinthala:** investigation, writing – review and editing, methodology, formal analysis. **Chelsea N. Monty‐Bromer:** conceptualization, funding acquisition, writing – review and editing, resources, supervision. **John M. Senko:** conceptualization, funding acquisition, writing – review and editing, supervision, resources, visualization.

## Conflicts of Interest

The authors declare no conflicts of interest.

## Data Availability

The data that support the findings of this study are openly available in the National Center for Biotechnology Information at https://www.ncbi.nlm.nih.gov, reference number PRJNA1058620.
